# Wavelet subband-specific learning for low-dose computed tomography denoising

**DOI:** 10.1371/journal.pone.0274308

**Published:** 2022-09-09

**Authors:** Wonjin Kim, Jaayeon Lee, Mihyun Kang, Jin Sung Kim, Jang-Hwan Choi

**Affiliations:** 1 Division of Mechanical and Biomedical Engineering, Graduate Program in System Health Science and Engineering, Ewha Womans University, Seoul, Republic of Korea; 2 Department of Cyber Security, Ewha Womans University, Seoul, Republic of Korea; 3 Department of Radiation Oncology, Yonsei University College of Medicine, Seoul, Republic of Korea; Korea National University of Transportation, KOREA, REPUBLIC OF

## Abstract

Deep neural networks have shown great improvements in low-dose computed tomography (CT) denoising. Early algorithms were primarily optimized to obtain an accurate image with low distortion between the denoised image and reference full-dose image at the cost of yielding an overly smoothed unrealistic CT image. Recent research has sought to preserve the fine details of denoised images with high perceptual quality, which has been accompanied by a decrease in objective quality due to a trade-off between perceptual quality and distortion. We pursue a network that can generate accurate and realistic CT images with high objective and perceptual quality within one network, achieving a better perception-distortion trade-off. To achieve this goal, we propose a stationary wavelet transform-assisted network employing the characteristics of high- and low-frequency domains of the wavelet transform and frequency subband-specific losses defined in the wavelet domain. We first introduce a stationary wavelet transform for the network training procedure. Then, we train the network using objective loss functions defined for high- and low-frequency domains to enhance the objective quality of the denoised CT image. With this network design, we train the network again after replacing the objective loss functions with perceptual loss functions in high- and low-frequency domains. As a result, we acquired denoised CT images with high perceptual quality using this strategy while minimizing the objective quality loss. We evaluated our algorithms on the phantom and clinical images, and the quantitative and qualitative results indicate that ours outperform the existing state-of-the-art algorithms in terms of objective and perceptual quality.

## 1 Introduction

X-ray computed tomography (CT) is widely used in many industries and is an essential clinical diagnostic tool. Moreover, it provides a noninvasive method of obtaining clinical information from patients. However, high radiation exposure is a concern in the use of CT. According to US statistics, the increased use of CT scans contributes to the potential risk of lung cancer [1]. Thus, a CT scan must be performed under the principle of as low as reasonably achievable [2]. Therefore, low-dose CT (LDCT) has been increasingly adopted. However, reducing CT radiation produces more noise in the CT scans; thus, research on LDCT denoising has been widely conducted in the medical imaging field.

In recent years, deep learning algorithms using a convolutional neural network (CNN) have demonstrated excellent performance compared to traditional machine learning algorithms in the computer vision community. This trend has also occurred in CT research, and LDCT denoising has benefited considerably from CNN denoising. An encoder-decoder CNN designed with a residual connection [3] was developed and proved that noise on the Mayo Clinic’s data can be removed effectively. Yang *et al*. [4] used 2D and 3D CNNs with residual networks. Kang *et al*. [5] provided an iterative framelet-based denoising algorithm.

Although these methods demonstrated successful results with high objective quality, the pixel-level loss based on the mean squared error (MSE) or mean absolute error (MAE) generated overly smoothed images with a significant loss in detailed texture and edges, which is not beneficial from the perspective of human visual perception [6]. Thus, after the early deep learning development of LDCT denoising, the current LDCT denoising goal has moved toward pursuing high perceptual quality to recover the details in denoised images.

VGG loss [7] and the generative adversarial network (GAN) [8] are commonly adopted when pursuing high perceptual quality in LDCT denoising. Badretale *et al*. [9] defined the loss function using the perceptual loss generated from the Visual Geometry Group (VGG) network [10] to better catch the details of texture and preserve the edges. Wolterink *et al*. [11] used the GAN for CT denoising, and Yi *et al*. [12] combined the conditional generative network and sharpness detection network to prevent blurring while denoising. The Wasserstein distance and perceptual loss were used through the GAN by Yang *et al*. [13]. You *et al*. [14] employed 3D volumetric information and perceptual loss with the GAN. Shan *et al*. [15] found that the 3D CNN has better results than the 2D CNN, and it can be trained by transfer-learning from the 2D trained network with GAN loss. Choi *et al*. [16] used statistical information and Li *et al*. [17] employed 3D self-attention to retrieve the denoised image with GAN loss.

To clarify the terminology in this paper, the low-distortion image referred to in the LDCT image denoising task indicates an image with high objective quality or a high value of the peak signal to noise ratio (PSNR). In addition, an image that preserves fine details or sharp edges that may provide important information for clinical diagnosis is referred to as a high perceptual image. High objective quality can be obtained when a network is trained with pixel-wise loss (MSE or MAE), which we call objective loss (*L*_*o*_). High perceptual quality can be achieved by optimizing the VGG loss (*L*_*vgg*_) or adversarial loss (*L*_*adv*_). If at least one of these losses is used to train a network, the network optimizes the perceptual loss (*L*_*p*_).

When an algorithm is trained based on perceptual loss, the resulting decrease in PSNR of the image can be explained as the perception-distortion (PD) trade-off, and a PD bound exists for image denoising algorithms [18]. Due to the PD trade-off phenomenon in the LDCT image denoising task, if we seek an image with a high PSNR, we obtain a blurred image that is inappropriate for clinical diagnostic use. In contrast, if we aim to achieve an image with high perception, we must be aware that the image noise increases and PSNR decreases.

**Fig 1:**
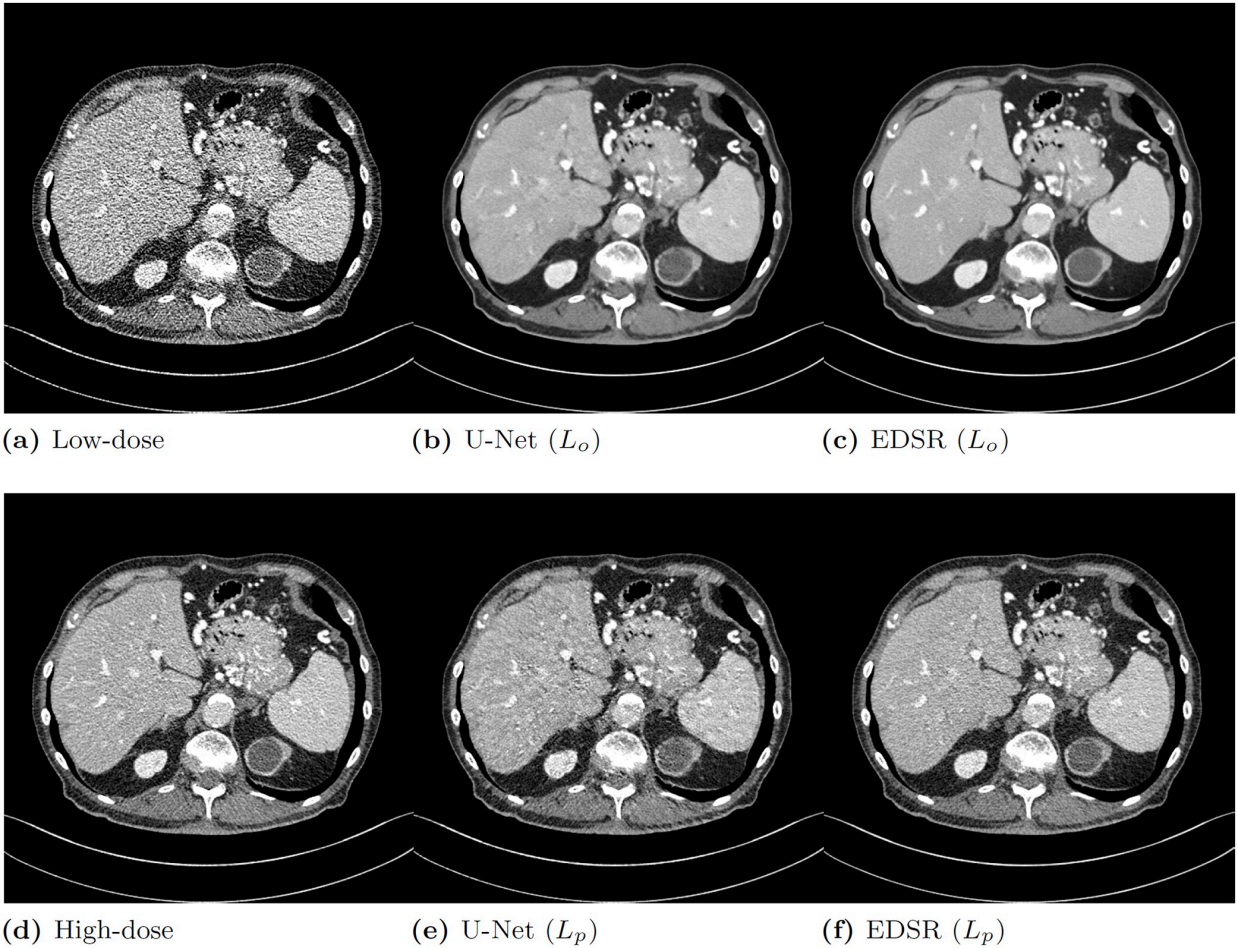
Representative examples of (a) the original low-dose computed tomography (CT) image, (d) normal-dose reference image, and denoised CT images from U-Net and EDSR optimized for objective loss (*L*_*o*_) (b) and (c) and perceptual loss (*L*_*p*_) (e) and (f).

To illustrate the PD trade-off with a visual example of LDCT, we optimized the representative image enhancement networks, U-Net [19] and EDSR [20], based only on either objective or perceptual loss. We compared their resulting images as depicted in Fig 1. The PSNR values corresponding to the denoised output images are summarized in Table 1. For EDSR, we added a global skip connection for LDCT denoising. Fig 1 and Table 1 reveal that the two network models based on perceptual loss obtained lower PSNR values than those based on objective loss (U-Net: 37.717 vs. 39.677 and EDSR: 38.856 vs. 39.877) but secured relatively high perceptual quality, showing sharper edges without losing details.

**Table 1:** Change in the peak signal to noise ratio (PSNR) value when U-Net and EDSR are optimized for perceptual loss (*L*_*p*_) or objective loss (*L*_*o*_). The networks had a lower PSNR value when optimized for perceptual loss than when optimized for objective loss.

Algorithms	U-Net (*L*_*o*_)	U-Net (*L*_*p*_)	EDSR (*L*_*o*_)	EDSR (*L*_*p*_)
PSNR	39.677	37.717	39.877	38.856

**Fig 2:**
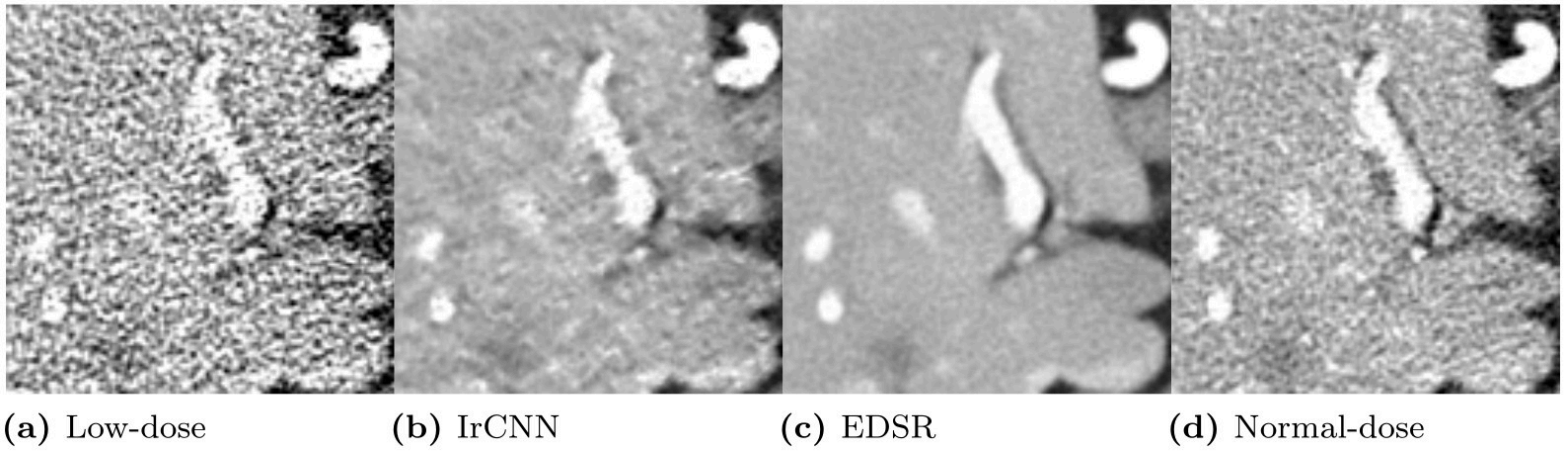
Close-up examples of (a) the original low-dose computed tomography (CT) image, (d) the normal-dose reference image, and noise-reduced CT image results from two different networks, (b) IrCNN (PSNR: 37.408) and (c) EDSR (PSNR: 39.875).

Although recent LDCT denoising studies by Yang *et al*. [13] and Shan *et al*. [15] have focused more on perceptual quality than the loss of objective quality, in our algorithm, we prioritize objective quality as highly as perceptual quality, which is expected to improve the PD bound because the problem of the low objective quality image is that it inherently has higher noise in the image. Fig 2 illustrates that IrCNN [21], which has a lower PSNR than EDSR [20], has poor noise reduction, thus, it makes harder to see details of structures. EDSR exhibited better noise reduction performance with a higher PSNR (*i.e*., higher objective quality) and better visibility than IrCNN although it resulted in an blurred image. In this example, we can see that high objective quality has also many advantages in denoising algorithms, thus, sacrificing high objective quality when seeking perceptual quality does not always have better results.

When we compare two identical networks, one maximizing objective quality and the other maximizing perceptual quality, the latter cannot exceed the objective quality of the former network. In other words, the maximum objective quality is determined by the network capability itself without considering any operation for perceptual quality. Thus, to obtain high objective and perceptual quality, we must first design a network that exhibits the best performance in objective quality when optimizing only objective loss. Then, with this network, we aim to secure the perceptual quality as much as possible while minimizing the loss of the objective quality.

The supervised learning-based LDCT denoising techniques have difficulty in obtaining perfectly paired low- and normal-dose images. Recently, unsupervised and semi-supervised learning algorithms have been developed for LDCT denoising to eliminate the needs of high-quality reference for training. Kim *et al*. [22] and Yuan *et al*. [23] provided a practical way to train the networks and generate both the training input and realistic label from the existing data with the help of physics-based CT noise model. Tang *et al*. [24] adopted CycleGAN [25] to train unpaired dataset for LDCT denoising. Although these methods have shown promising results, their performance is still inferior to that of supervised learning [26] and they have difficulty in preserving fine anatomical details due to their simple noise model [27].

In this paper, we suggested a novel LDCT denoising strategy based on the wavelet transform to enhance both objective and perceptual quality. The wavelet transform has been used in several studies on deep learning-based image denoising [5, 28, 29]. However, none of these studies have taken full advantage of the strength of the wavelet properties for better objective and perceptual quality. All previous studies have used the wavelet transform only as input or an operation of one layer but never used the frequency properties.

The wavelet transform can decompose a signal into high- and low-frequency subbands with their own properties. The low-frequency subband is responsible for the overall objective quality, whereas high-frequency subbands are very sensitive to small changes in fine details and substantially influence the perceptual quality. We employed the characteristics of high- and low-frequency subbands of the wavelet transform and defined the losses in the wavelet domain. With this wavelet domain loss, we minimized the loss of the objective quality when seeking perceptual quality in one network.

The main contributions of this paper are as follows:

- A stationary wavelet transform-assisted network is proposed to perform the LDCT image denoising task using newly defined wavelet losses in low and high frequency wavelet subbands. The network achieved the highest objective quality in LDCT denoising compared to the current state-of-the-art denoising algorithms for natural RGB and LDCT images.
- We also proposed a novel wavelet subband-specific learning strategy that allows our method to recover high perceptual quality with less compensation for high objective quality. Our method achieved competitive perceptual quality with the highest objective quality compared to the current state-of-the-art LDCT denoising methods.
- Our extensive experiments on real datasets (*in vivo* and phantom data) reveal that the proposed methods convincingly improve the denoising performance with a better PD trade-off over the existing state-of-the-art algorithms.

## 2 Method

### 2.1 Overall architecture

**Fig 3:**
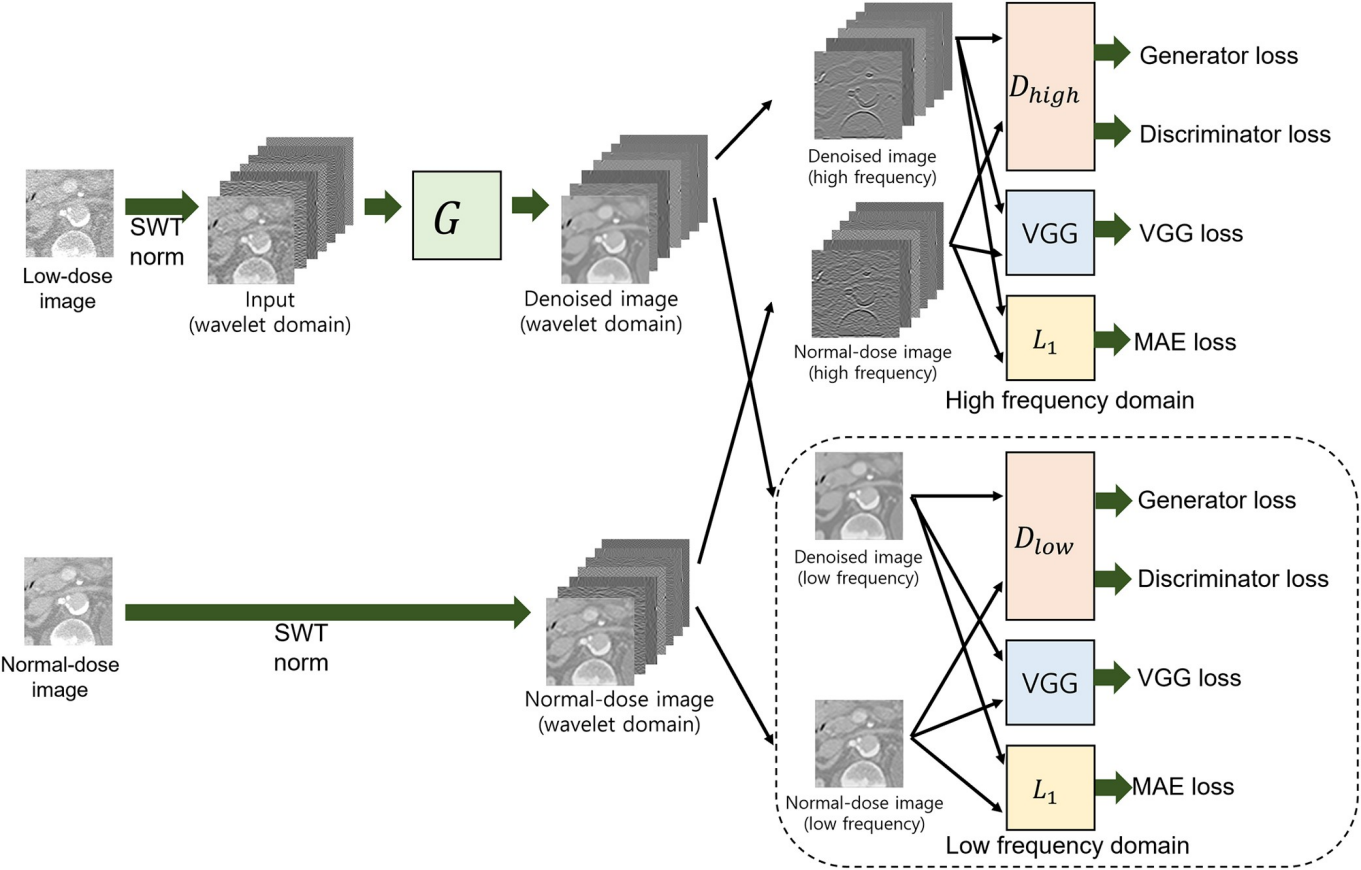
Overall architecture of the proposed methods, where *G* is a generator (denoiser). *D*_*high*_ and *D*_*low*_ are discriminators in high- and low-frequency domains, VGG loss used VGG19 [10] and *L*_1_ loss is the mean absolute error (MAE), each of which calculates the loss between entered two inputs of denoised and ground truth clean images. The variables *x*, $\tilde{y}$, and *y* denote the noisy input CT image, denoised CT output image, and clean CT image. Further, *wx*, $w\tilde{y}$ and *wy* are denoted as *x*, $\tilde{y}$ and *y* in the SWT domain with normalization in each subband. From $w\tilde{y}$ and *wy*, $w{\tilde{y}}_{low}$ and *wy*_*low*_ is the low-frequency subband, and $w{\tilde{y}}_{high}$ and *wy*_*high*_ includes the high frequency subbands.

Fig 3 displays the overall architecture of the proposed method. The network is based on EDSR [20], but we modified the network by adding a global skip connection for denoising. After building the base network, we adopted a stationary wavelet transform (SWT) to enhance the objective quality further. We applied a level 2 SWT to decompose the input noisy LDCT images into seven different frequency subbands. Then, we normalized each subband and used them as input to the network. We defined the wavelet loss to optimize the objective loss with the network output in the wavelet domain and secured the maximum objective quality first with our proposed network.

Finally, to obtain denoised images with high perceptual quality, we redefined the loss function in the wavelet domain by introducing perceptual loss, the VGG and adversarial losses. The purpose of the redefined loss function in the wavelet domain was to increase the perceptual quality while maintaining the objective quality maximized in the previous step. To achieve this goal, we assigned different weights for each loss in high- and low-frequency subbands to use the characteristics of high- and low-frequency image components. In low-frequency domain loss, we assigned more weight to the objective loss term to increase the objective quality, whereas we assigned more weight to perceptual loss terms for high-frequency domain loss, enhancing the perceptual quality. The relevant details are described in the following subsections.

### 2.2 Generator and discriminator

**Fig 4:**
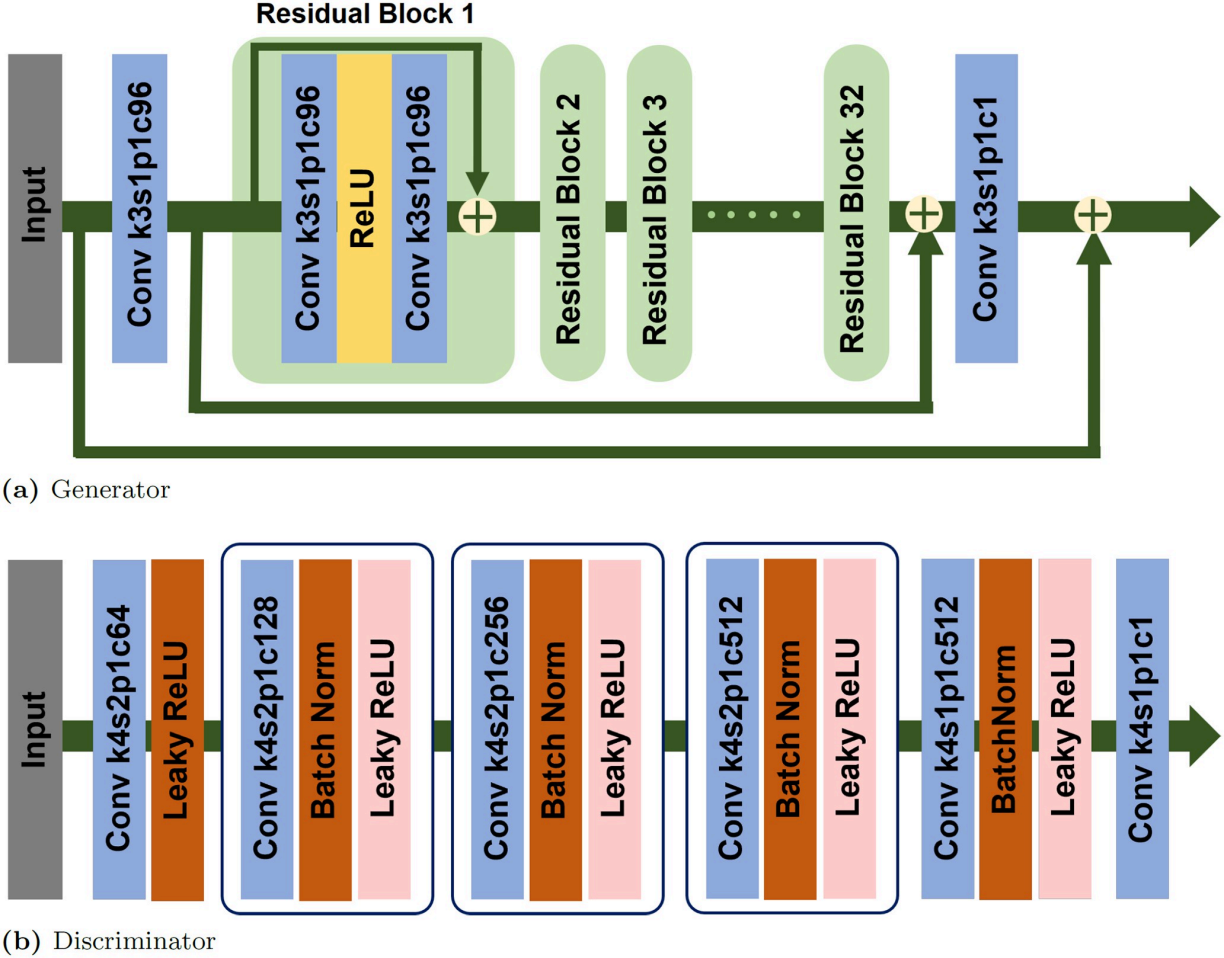
Architecture of the generator and discriminator. In convolution operator, k, s, p, and c stand for kernel, stride, padding, and the number of output channels.

Generator and discriminator networks are depicted in Fig 4. The generator, used as a denoiser, consists of one convolution for feature extraction, 32 residual blocks, and a final block convolution for image reconstruction. Each residual block has convolution, ReLU, and convolution sequentially. Moreover, each convolution is defined with 3 × 3 kernel size, 1 stride, 1 padding, and 96 channels. The global skip connection is used to learn the residuals, such as a DnCNN [30]. We have two discriminators for high- and low-frequency domains. The network architecture of the discriminator is based on PatchGAN [31].

### 2.3 Stationary wavelet transform and subband analysis

A wavelet transform decomposes a signal into a set of basis functions consisting of contractions, expansions, and translations of a mother function, called the wavelet, enabling multiresolution image analysis [32]. The classical discrete wavelet transform (DWT) usually decomposes the original image into a sequence of new images with decreased size, and the SWT decomposes a signal into new images with the same size as the original image. Both the DWT and SWT have the advantage of expanding their receptive fields because of downsampling in the DWT or upsampling the convolutional filter in the SWT.

The SWT overcomes the drawback of the DWT, which is not shift-invariant. Moreover, using the SWT enables us to build better networks that achieve higher objective quality performance than the U-Net or encoder-decoder architecture with the DWT adopted [28]. Thus, although previous wavelet-based image denoising studies used the DWT [28, 29], we used the SWT, considering the relative advantages of the SWT.

**Fig 5:**
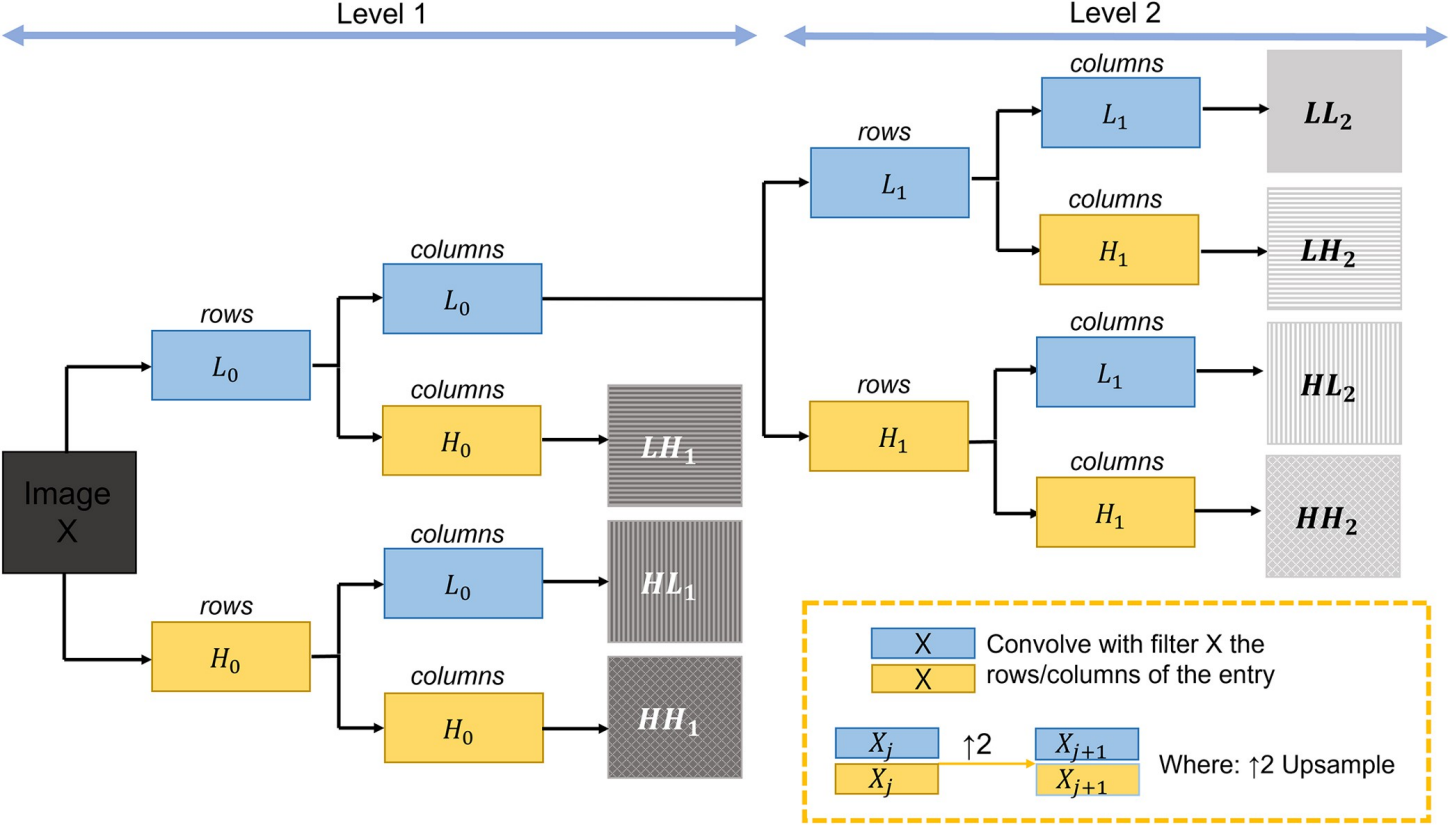
Two-level stationary wavelet transform of image *X*, with *L*_0_ and *H*_0_ as the specified lowpass and highpass wavelet decomposition filters.

The SWT is implemented using the filter-bank algorithm, which is depicted in Fig 5. We used the Haar function as our wavelet function. Let *h* and *g* be the scaling and wavelet filter, respectively. Then, the SWT of the scaling filter and wavelet filter at scale *j* + 1 is defined recursively as follows:

$$\begin{array}{c} h_{j+1}\left[k\right]=h_{j}\left[k\right]⇑2=\left\{ \begin{array}{cc} h_{j}[\frac{k}{2}] & \text{,}\;\text{k}\;\text{is}\;\text{even}\\ 0 & \text{,}\;\text{k}\;\text{is}\;\text{odd} \end{array}\right. \end{array}$$ (1)

$$\begin{array}{c} g_{j+1}\left[k\right]=g_{j}\left[k\right]⇑2=\left\{ \begin{array}{cc} g_{j}[\frac{k}{2}] & \text{,}\;\text{k}\;\text{is}\;\text{even}\\ 0 & \text{,}\;\text{k}\;\text{is}\;\text{odd} \end{array}\right. \end{array}$$ (2)

where *h*_0_[*k*] = *h*[*k*] and *g*_0_[*k*] = *g*[*k*]. The *J*th level SWT of an image *x* is then calculated recursively with the filter-bank operations.

$$\begin{array}{c} c_{j+1}=h_{j}\left[-k\right]*c_{j}\left[k\right] \end{array}$$ (3)

$$\begin{array}{c} d_{j+1}=g_{j}\left[-k\right]*c_{j}\left[k\right] \end{array}$$ (4)

where *c*_0_ = *x*, *j* = 0, …, *J* − 1, , and * is the convolution operation.

In Fig 5, the SWT at level 2 decomposes a one-channel image into seven subbands, thus, one subband (*LL*_2_) includes low-frequency information, and the other six subbands (*LH*_2_, *HL*_2_, *HH*_2_, *LH*_1_, *HL*_1_, and *HH*_1_) contain high-frequency information.

**Fig 6:**
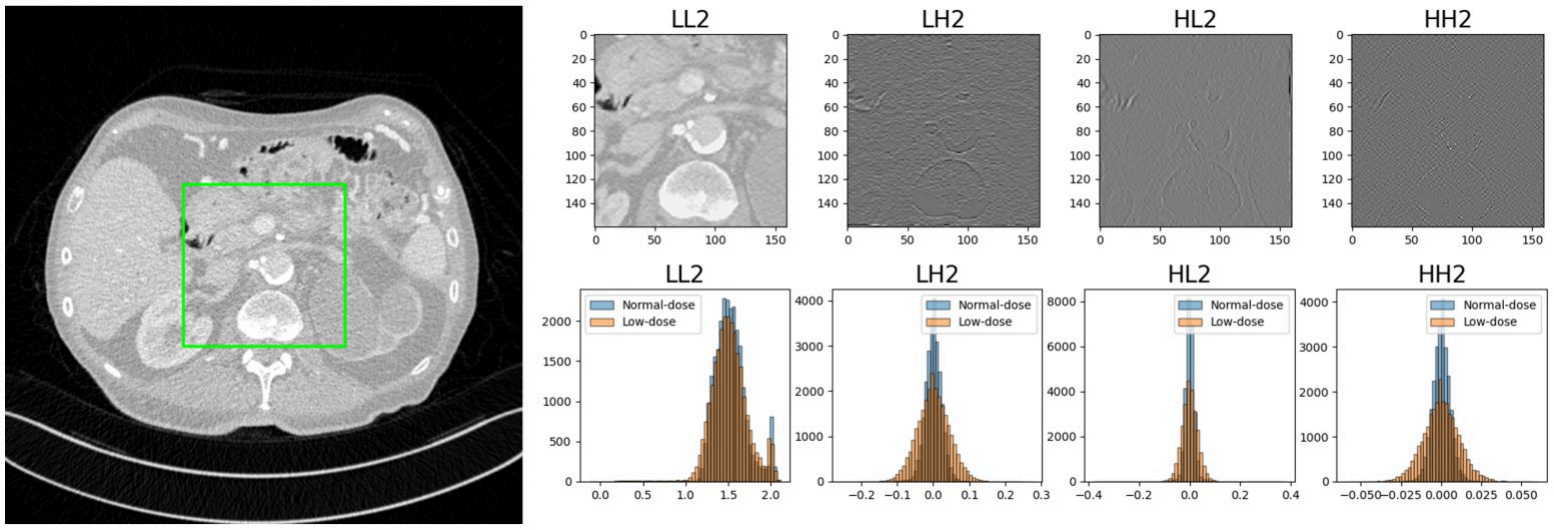
Example of a stationary wavelet transform (above) and comparison of the histogram distribution (below) of low-dose and normal-dose (reference) CT images in stationary wavelet transform domain. For better visuality, we included only *LL*_2_, *LH*_2_, *HL*_2_, and *HH*_2_ subbands. Four different images correspond to histograms for the low-frequency (*LL*_2_) and high-frequency subbands (*LH*_2_, *HL*_2_, and *HH*_2_).

Since the low-frequency subband *LL*_2_ contains most of the energy (*i.e*., the overall shape) of the original image as depicted in Fig 6, it plays a more dominant role than other high-frequency subbands in determining the objective image quality. However, high-frequency subbands are as important as low-frequency subbands because the high-frequency subbands present textural details and differences in the objective quality of the state-of-the-art algorithms are very small. Thus, we managed high-frequency subbands carefully by determining the best combination of the weights for each frequency subband to maximize the objective quality.

Comparing the histograms of low-dose and normal-dose images in Fig 6 reveals that their distributions are similar in low-frequency subbands but different in high-frequency subbands, which implies that we should manage high-frequency subbands more precisely. When we minimize objective loss functions, we can increase our objective quality. However, this optimization process tends to make distributions of high-frequency subbands centered on 0. As a result, the network that optimizes the objective loss function yields an overly smoothed denoised image with lost detailed information. With this observation, we must alleviate this zero-centered distribution with new defined perceptual losses. In our approach, we divided loss functions into high- and low-frequency domains and defined each of them according to the frequency characteristics. We focused more on objective quality in the low-frequency domain and tried to enhance perceptual quality in the high-frequency domain.

### 2.4 Frequency subband-specific loss on the wavelet transform domain

In order to maximize the objective and perceptual quality, we took a strategy to secure high objective quality first and minimize the loss of objective quality while pursuing perceptual quality, and we defined the necessary loss in the wavelet transform domain as follows.

The variables *x*, $\tilde{y}$, and *y* denote the noisy input CT image, denoised CT output image, and clean CT image. Further, *wx*, $w\tilde{y}$ and *wy* are denoted as *x*, $\tilde{y}$ and *y* in the SWT domain with normalization in each subband. Moreover, *G* is a generator and can be a denoiser as well. Thus, we can formulate the following:

$$\begin{array}{c} wx=norm(swt(x)) \end{array}$$ (5)

$$\begin{array}{c} wy=norm(swt(y)) \end{array}$$ (6)

$$\begin{array}{c} w\tilde{y}=G\left(wx\right) \end{array}$$ (7)

To accomplish high objective quality, we first define the objective loss in the low- and high-frequency domains in the wavelet transform domain as follows:

$$\begin{array}{c} L_{ho}=\frac{1}{wh}\left|w{\tilde{y}}_{high}-wy_{high}\right| \end{array}$$ (8)

$$\begin{array}{c} L_{lo}=\frac{1}{wh}\left|w{\tilde{y}}_{low}-wy_{low}\right| \end{array}$$ (9)

where *w* and *h* are the width and height of the image, *low* is the one channel subband of *LL*_2_, and *high* includes the *LH*_2_, *HL*_2_, *HH*_2_, *LH*_1_, *HL*_1_, and *HH*_1_ subbands. Then, we define the total objective loss by combining *L*_*lo*_ and *L*_*ho*_.

$$\begin{array}{c} L_{wo}=\alpha_{low}L_{lo}+\left(1-\alpha_{low}\right)L_{ho} \end{array}$$ (10)

where *α*_*low*_ is a hyper-parameter and controls the weight of the low-frequency subband. With the proper parameter, optimizing *L*_*wo*_ with our proposed network achieves the best performance in objective quality compared to the existing algorithms.

We defined the VGG loss to pursue perceptual quality as follows:

$$\begin{array}{c} L_{hvgg}=\frac{1}{w_{f}h_{f}}\left|VGG\left(GM\left(w{\tilde{y}}_{high}\right)\right)-VGG\left(GM\left(wy_{high}\right)\right)\right| \end{array}$$ (11)

$$\begin{array}{c} L_{lvgg}=\frac{1}{w_{f}h_{f}}\left|VGG\left(GM\left(w{\tilde{y}}_{low}\right)\right)-VGG\left(GM\left(wy_{low}\right)\right)\right| \end{array}$$ (12)

The *VGG* operation used VGG-19 [10] to extract the feature maps at the second convolutional layer after the second maxpool operation, called ReLU2_2. We applied the Gram matrix to the feature map from VGG-19, which is denoted as *GM*. In addition, *w*_*f*_ and *h*_*f*_ are the width and height of feature map after the Gram matrix output.

Adversarial loss is defined in both the low- and high-frequency domains:

$$\begin{array}{c} L_{hadv}=E_{wx_{high}∼p_{data}\left(wx_{high}\right)}\left[log\left(1-D_{high}\left(w{\tilde{y}}_{high}\right)\right)\right] \end{array}$$ (13)

$$\begin{array}{c} L_{ladv}=E_{wx_{low}∼p_{data}(wx_{low})}\left[log\left(1-D_{low}\left(w{\tilde{y}}_{low}\right)\right)\right] \end{array}$$ (14)

where *D*_*high*_ and *D*_*low*_ are the discriminators for the high- and low-frequency domains respectively. By introducing adversarial loss, we can defined the GAN network [8] to optimize the following:

$$\begin{array}{l} min_{G}max_{D_{high}}L_{hGAN}=E_{y_{high}\sim p_{data}(y_{high})}[logD_{high}(wy_{high})]+\\ E_{x_{high}\sim p_{data}(x_{high})}[log(1-D_{high}(w{\tilde{y}}_{high}))] \end{array}$$ (15)

$$\begin{array}{l} min_{G}max_{D_{low}}L_{lGAN}=E_{y_{low}\sim p_{data}(y_{low})}[logD_{low}(wy_{low})]+\\ E_{x_{low}\sim p_{data}(x_{low})}[log(1-D_{low}(w{\tilde{y}}_{low}))] \end{array}$$ (16)

We combined all losses in the high- and low-frequency domains with the objective loss in the wavelet domain for perceptual quality:

$$\begin{array}{c} L_{hp}=\alpha_{ho}L_{ho}+\alpha_{hvgg}L_{hvgg}+\alpha_{hgan}L_{hGAN} \end{array}$$ (17)

$$\begin{array}{c} L_{lp}=\alpha_{lo}L_{lo}+\alpha_{lvgg}L_{lvgg}+\alpha_{lgan}L_{lGAN} \end{array}$$ (18)

Then, the total loss is redefined in the same way as in *L*_*wo*_:

$$\begin{array}{c} L_{wp}=\alpha_{low}L_{lp}+\left(1-\alpha_{low}\right)L_{hp} \end{array}$$ (19)

where *α*_*low*_ is the same value from (10).

*α*_*ho*_, *α*_*hvgg*_, and *α*_*hGAN*_ are the high-frequency objective weight, VGG loss weight, and GAN loss weight, and *α*_*lo*_, *α*_*lvgg*_, and *α*_*lGAN*_ are the low-frequency objective weight, VGG loss weight, and GAN loss weight respectively in the wavelet loss domain. These hyperparameters control the importance of each loss. In the low-frequency domain, we set *α*_*lo*_, *α*_*lvgg*_, and *α*_*lGAN*_ as 1.0, 0.1 and 0.0001. We assigned a high weight to the objective loss and less weight to the perceptual loss terms in the low-frequency domain. Moreover, *α*_*lvgg*_ is set as 0.1 because it also contains textures and edges, even in the low-frequency domain.

In the high-frequency domain, we set *α*_*ho*_, *α*_*hvgg*_, and *α*_*hGAN*_ as 0, 1.0, and 0.01, respectively. We did not add objective loss in the high-frequency domain and assigned higher weights for the VGG and GAN loss than in the low-frequency domain. Thus, we maintained the detailed information of the denoised image while minimizing the loss of the objective quality. We evaluated the performance of our proposed model optimized for both *L*_*wo*_ and *L*_*wp*_ loss functions.

Loss functions for objective and perceptual losses, which are commonly adopted for other algorithms, are defined similarly to *L*_*wo*_ and *L*_*wp*_, but without the SWT operation:

$$\begin{array}{c} L_{o}=\frac{1}{wh}\left|\tilde{y}-y\right| \end{array}$$ (20)

$$\begin{array}{c} L_{p}=\alpha_{vgg}L_{vgg}+\alpha_{gan}L_{GAN} \end{array}$$ (21)

We used these loss functions for the network based on the U-Net and EDSR and *α*_*vgg*_ and *α*_*gan*_ as 1.0 and 0.001, respectively, to compare results.

### 2.5 Normalization of wavelet subbands

We normalized each wavelet subband using the mean and standard deviation and updated them in the same way by updating the mean and standard deviation in the batch normalization [33]. First we calculated the batch mean (*μ*_*B*_) and standard deviation (*σ*_*B*_) as follows:

$$\begin{array}{c} \mu_{B}=\frac{1}{m}\sum_{i=1}^{m}x_{i} \end{array}$$ (22)

$$\begin{array}{c} \sigma_{B}^{2}=\frac{1}{m}\sum_{i=1}^{m}{\left(x_{i}-\mu_{B}\right)}^{2} \end{array}$$ (23)

where *m* is the number of training samples for a mini-batch. Then, we updated the mean and variance for normalization:

$$\begin{array}{c} E\left[x^{\left(k\right)}\right]=E_{B}\left[\mu_{B}^{\left(k\right)}\right] \end{array}$$ (24)

$$\begin{array}{c} Var\left[x^{\left(k\right)}\right]=\frac{m}{m-1}E_{B}\left[{\sigma_{B}^{\left(k\right)}}^{2}\right] \end{array}$$ (25)

We updated the mean and variance for 10,000 iterations, and afterward, we froze them.

The role of normalization of wavelet subbands is optimizing every subband equally by balancing the weights of each subband. As depicted in Fig 6, the low-frequency (*LL*_2_) subband has relatively high coefficients, thus, the loss in low frequency is relatively high compared to other high-frequency losses. By normalizing each subband, we can readjust the subband coefficients with the normal distribution and evenly assign the weights of each subband in the wavelet domain loss. Without normalization, low frequency subband, which has large energy, has higher prior to optimize than high frequency subbands, thus objective quality was slightly decreased.

### 2.6 Experimental Setup

#### 2.6.1 In vivo and phantom data acquisition

We scanned the anthropomorphic phantoms of the chest, neck, and pelvis and a Catphan 500 phantom [34] on two different multislice CT scanners (Siemens SOMATOM Sensation Open and Toshiba Aquilion TSX-201A). Table 2 lists the acquisition protocols used to obtain the image for the phantom datasets. A fixed tube voltage (120 kV) was used in all images. After acquiring a normal-dose image using routine CT acquisition protocols for each organ used in the clinic, a low-dose image pair was acquired with a low tube current exposure time product (mAs) value about 25% of normal-dose image. We divided training and test dataset within the volume of each phantom object. We used the lower 90% axial slices in the z-axis direction of the volume for model training and validation, and the remaining upper 10% for testing. The independent test dataset was not utilized during the model training or validation phase.

**Table 2:** Computed tomography acquisition parameters used to acquire normal-dose (high), low-dose (low) image pairs of anthropomorphic phantoms and a quality assurance phantom. The reported radiation dose values are in mAs units, and # represents the number of CT axial slice images.

	Siemens			Toshiba		
	#	High	Low	#	High	Low
pelvis	233	200	50	375	200	50
head-neck	188	180	40	283	180	30
chest	340	180	30	515	180	30
Catphan	159	150	30	242	150	30

For clinical data, we used the Mayo Clinic dataset (2016 NIH-AAPM-Mayo Clinic Low Dose CT Grand Challenge) [35]. These clinical data were obtained after approval by the institutional review board of Mayo clinic. The library was HIPAA compliant and built with a waiver of informed consent. The Mayo Clinic dataset contains anonymized CT images of ten patients in total. Each patient record contains normal-dose abdominal CT images and quarter-dose CT images. There are 1 mm and 3 mm thicknesses in the dataset, and we used both thicknesses for our training and testing. We first chose CT slices of three patients for test dataset, which include more number of lesions with small shape and can be regarded as clinically difficult task for diagnosis. CT slices of other seven patients were used for training and validation. We divided the training and validation of the CT slices of seven patients randomly with a ratio of 0.95:0.05.

#### 2.6.2 Experimental setting

For all the experiments, we used the Adam solver [36]. All networks were trained with a learning rate of 0.0002. We scheduled the learning rate to halve when the minimum loss does not change after five iterations. All images were normalized between 0 and 1 and were used as input for the proposed method. Data augmentation is performed on training images, including random rotations of 90, 180, and 270 and flipping horizontally. In each training batch, a random patch with a size of 80 × 80 is extracted as input. The networks are implemented in the PyTorch framework and trained with four Nvidia Tesla V100 graphical processing units. We set the same settings except that images are normalized between -0.5 and 0.5 when we implemented the existing state-of-the-art algorithms to compare the performance.

## 3 Experimental results

### 3.1 Ablation study

#### 3.1.1 Effectiveness of designing the network

Our proposed network design was modified from EDSR, and its performance was improved through the following structural modifications. A global skip connection allows the network to learn the residual, which was introduced in the DnCNN [30], thus enabling the network to learn the image noise. Therefore, the global skip connection has been adopted in denoising algorithms in [28, 37]. Then, the network performance was enhanced by replacing the CT image input with the SWT seven-channel subbands. Finally, we adopted normalization to increase the network performance.

The performance increase in the PSNR by gradually adding each feature was summarized in Table 3, demonstrating that the strategy effectively achieved higher objective quality in the network design.

**Table 3:** The EDSR-based model’s performance improvement, benefiting from gradual structural modification.

	EDSR	+global skip	+SWT	+normalization
PSNR	39.875	39.896	39.939	39.950

#### 3.1.2 Weight of a low-frequency subband in wavelet domain loss

We evaluated the objective image quality by varying the low-frequency weight *α*_*low*_ in (10) by 0.15 from 0.2 to 0.8, and the resulting PSNR value is reported in Table 4. We did not include *α*_*low*_ of 0 and 1.0 because the resulting PSNR values are very low, 8.403 and 28.401, respectively. As indicated in Table 4, by assigning low-frequency weight as 0.2 and high-frequency weight as 0.8 (1-*α*_*low*_), which mean we strengthen the high-frequency domain loss more than the low-frequency domain loss, we gain the best performance of objective quality with the proposed network. We applied this obtained *α*_*low*_ (0.2) to (19) and trained the network to secure a high PSNR while pursuing perceptual quality.

**Table 4:** Changes in objective image quality in terms of the peak signal to noise ratio (PSNR) depending on the low-frequency weight value (*α*_*low*_) when optimizing *L*_*wo*_ loss.

*α*_*low*_	0.2	0.35	0.5	0.65	0.8
PSNR	39.950	39.942	39.940	39.904	39.845

### 3.2 Denoising results

#### 3.2.1 Histogram distribution results

**Fig 7:**
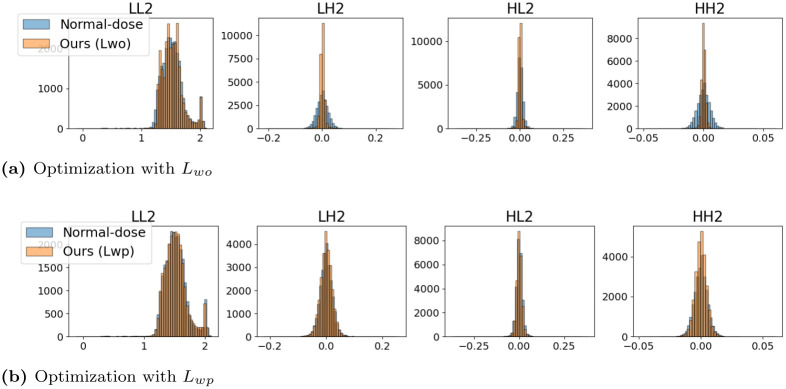
Histogram distribution results from two networks that optimized *L*_*wo*_ and *L*_*wp*_. For better visuality, we included only *LL*_2_, *LH*_2_, *HL*_2_, and *HH*_2_ subbands.

We tested how the histogram distribution changes using the proposed algorithms in Fig 7. When we optimized the objective losses with *L*_*wo*_, the histogram distribution is zero-centered in high-frequency subbands; thus, the denoised images have less detailed information. However, in terms of objective quality, the zero-centered distribution is the average subband value, which coincides with the fact that the denoised image from the denoising network is the average output of all plausible images [38].

In contrast, when we optimized the perceptual loss with *L*_*wp*_, the histogram distribution of the denoised images in the high-frequency subbands demonstrates more comparable matching between normal-dose and denoised CT images. Thus, the denoised CT images have richer textures and patterns, which look like the ground truth CT images, whereas it might entail the loss of objective image quality.

#### 3.2.2 Objective quality results

We compared the proposed method with the existing state-of-the-art image denoising algorithms for RGB natural and LDCT images that maximize the objective quality using the Mayo Clinic dataset to validate the network effectiveness with the optimization of *L*_*wo*_. In natural RGB image denoising algorithms, it is still common to maximize only the objective quality, so we compared our algorithm with the optimization of *L*_*wo*_ to the image denoising algorithms. For fair comparison, all algorithms were trained on Mayo Clinic dataset. As summarized in Table 5, our proposed network with the optimization of *L*_*wo*_ performed the best in terms of PSNR compared with other existing state-of-the-art denoising algorithms for natural RGB images and LDCT images.

**Table 5:** Comparisons of the objective quality with state-of-the-art algorithms for RGB and LDCT images.

Algorithms	PSNR	Algorithms	PSNR
MLP [39]	26.700	WavResNet [5]	39.692
U-Net [19]	39.677	RCAN [40]	39.890
RED30 [41]	39.594	CBDNet [42]	38.352
IrCNN [21]	37.408	RIDNet [43]	39.723
DnCNN [30]	39.481	DHDN [44]	39.664
MemNet [45]	39.727	BRDNet [46]	39.864
RED-CNN [3]	39.613	RDN [37]	39.483
FFDNet [47]	39.248	DSWN [29]	39.881
MWCNN [28]	39.812	**Ours (*L*_*o*_)**	**39.950**

In addition, we analyzed whether our proposed network maintains a better objective quality in the process of optimizing both objective (*L*_*wo*_) and perceptual loss (*L*_*wp*_) compared with the following existing LDCT denoising algorithms: U-Net [19], RED-CNN [3], WavResNet [5], WGAN-VGG [13], and CPCE3D [15]. The original U-Net is for segmentation tasks, but with a slight modification, it has also been widely used in denoising tasks [6]. When implementing WavResNet, instead of using the contourlet transform, we used the SWT with the Haar function. While implementing WGAN-VGG, U-Net replaced the original generator because it makes the network more stable when optimizing the network.

We can divide these LDCT denoising algorithms into two groups: one group with U-Net [19], RED-CNN [3], WavResNet [5], and our network with optimization *L*_*wo*_, and the other group with WGAN-VGG [13], CPCE3D [15], and our network with optimization *L*_*wp*_. The former group maximized the objective quality by optimizing the objective loss, and the latter group pursued the perceptual quality by optimizing the perceptual loss. As presented in Table 6, our two proposed networks (optimizing *L*_*wo*_ and *L*_*wp*_) have a higher objective quality than others in each group in both the PSNR and structural similarity index measure (SSIM) metrics.

**Table 6:** Comparison of the objective quality for phantom and Mayo Clinic datasets.

Optimization	Algorithm	Phantom		*In vivo* clinic	
		PSNR	SSIM	PSNR	SSIM
Objective loss	RED-CNN	42.852	0.9771	39.613	0.9867
	WavResNet	43.050	0.9776	39.692	0.9849
	U-Net	44.227	0.9776	39.677	0.9877
	Ours (*L*_*wo*_)	44.659	0.9785	39.950	0.9878
Perceptual loss	WGAN-VGG	41.925	0.9700	38.478	0.9838
	CPCE3D	41.598	0.9713	38.517	0.9847
	Ours (*L*_*wp*_)	42.997	0.9745	39.167	0.9862

#### 3.2.3 Perceptual quality results

**Fig 8:**
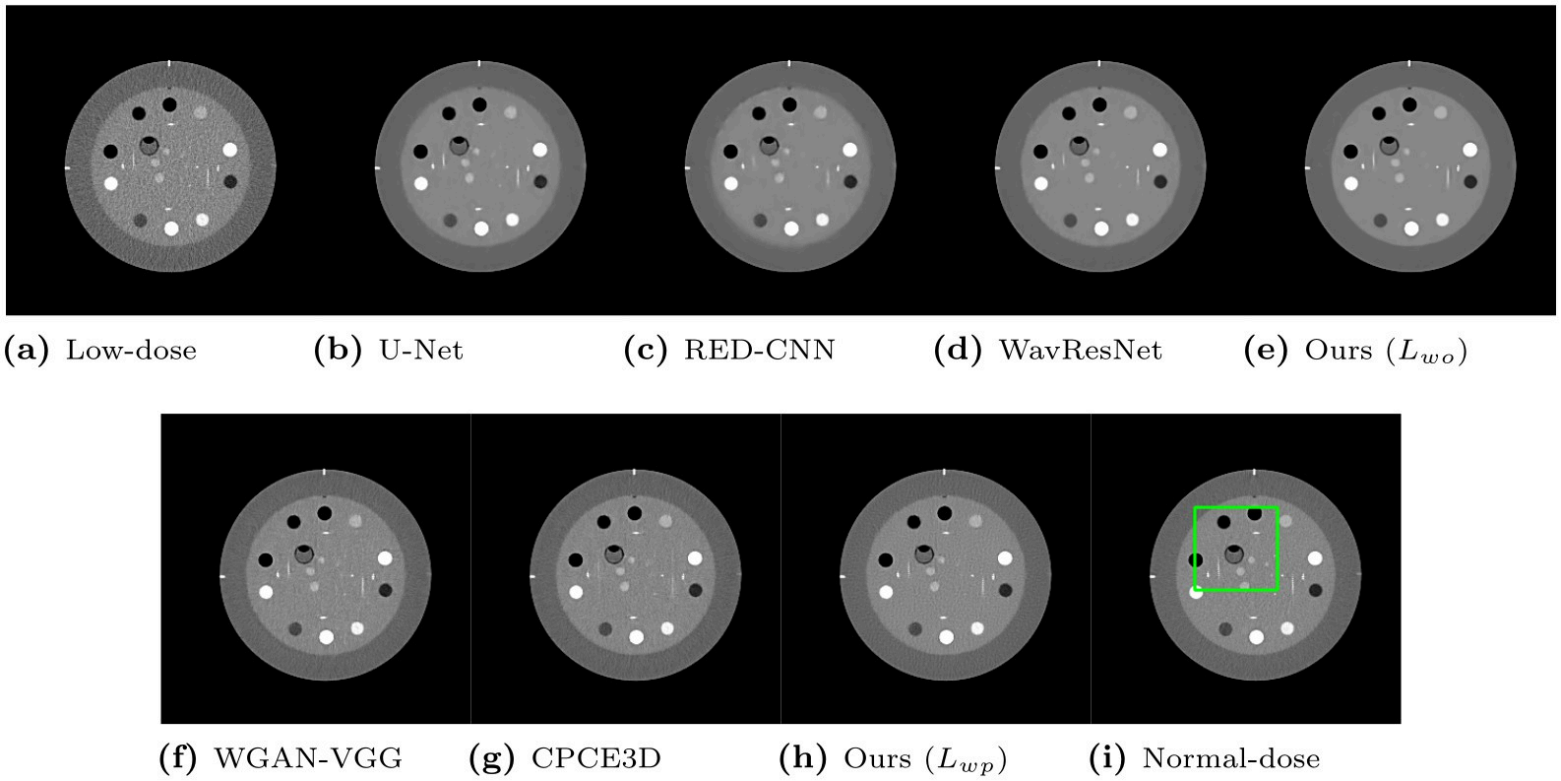
Representative slice from the Catphan object in the phantom dataset. The display window is [-160, 240]HU.

**Fig 9:**
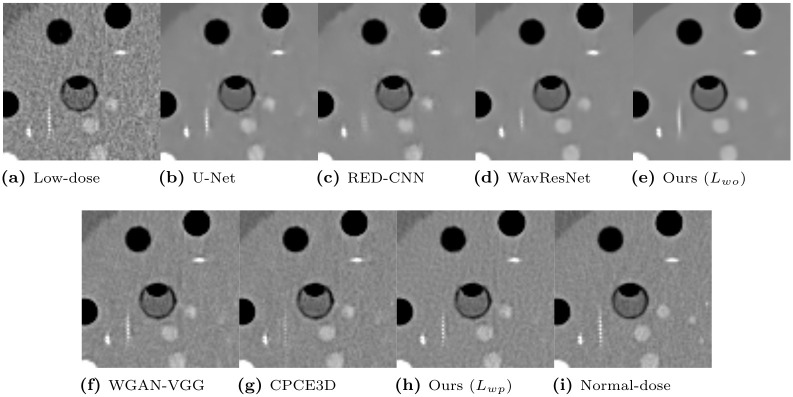
Regions of interest from Fig 8. The display window is [-160, 240]HU.

To qualitatively compare the perceptual image quality, we selected several CT images. Window levels in Hounsfield unit (HU) are adjusted and written in figures. Fig 8 presents representative denoised output slices of the Catphan phantom. For a clearer visual comparison between the resulting images, their close-up images are also displayed in Fig 9. Our proposed method of optimizing *L*_*wo*_ has the minimum noise among all algorithms, revealing smooth surface over piece-wise constant regions with the same density. If we compare our method with *L*_*wo*_ (Fig 9(e)) to the networks optimizing the objective loss in Fig 9(b) to 9(d), it has less noise in the denoised output and the shape of the objects are better kept than the others. However, textural details are lost because the output results present blurry images. For instance, the linearly aligned dots in Fig 9(e) cannot be distinguished from each other.

In contrast, the networks optimizing the perceptual loss in Fig 9(f) to 9(h) preserve these shapes and edges better than the networks optimizing the objective loss in Fig 9(b) to 9(e). Our proposed algorithm with *L*_*wp*_ (Fig 9(h)) preserves the detailed structures better than other algorithms optimizing perceptual loss. For instance, Fig 9(h) exhibits clearer separation and accurate placement of the vertically aligned points than the other, thus, our algorithm outputs more reliable and realistic CT results. Among the compared algorithms optimizing perceptual loss, the proposed algorithm with *L*_*wp*_ had the highest quantitative PSNR value, as displayed in Table 6. The proposed algorithm exhibited the best qualitative noise reduction performance. Therefore, the proposed algorithm, which optimized *L*_*wp*_ demonstrates the effectiveness of reducing noise and preserving information with phantom datasets.

**Fig 10:**
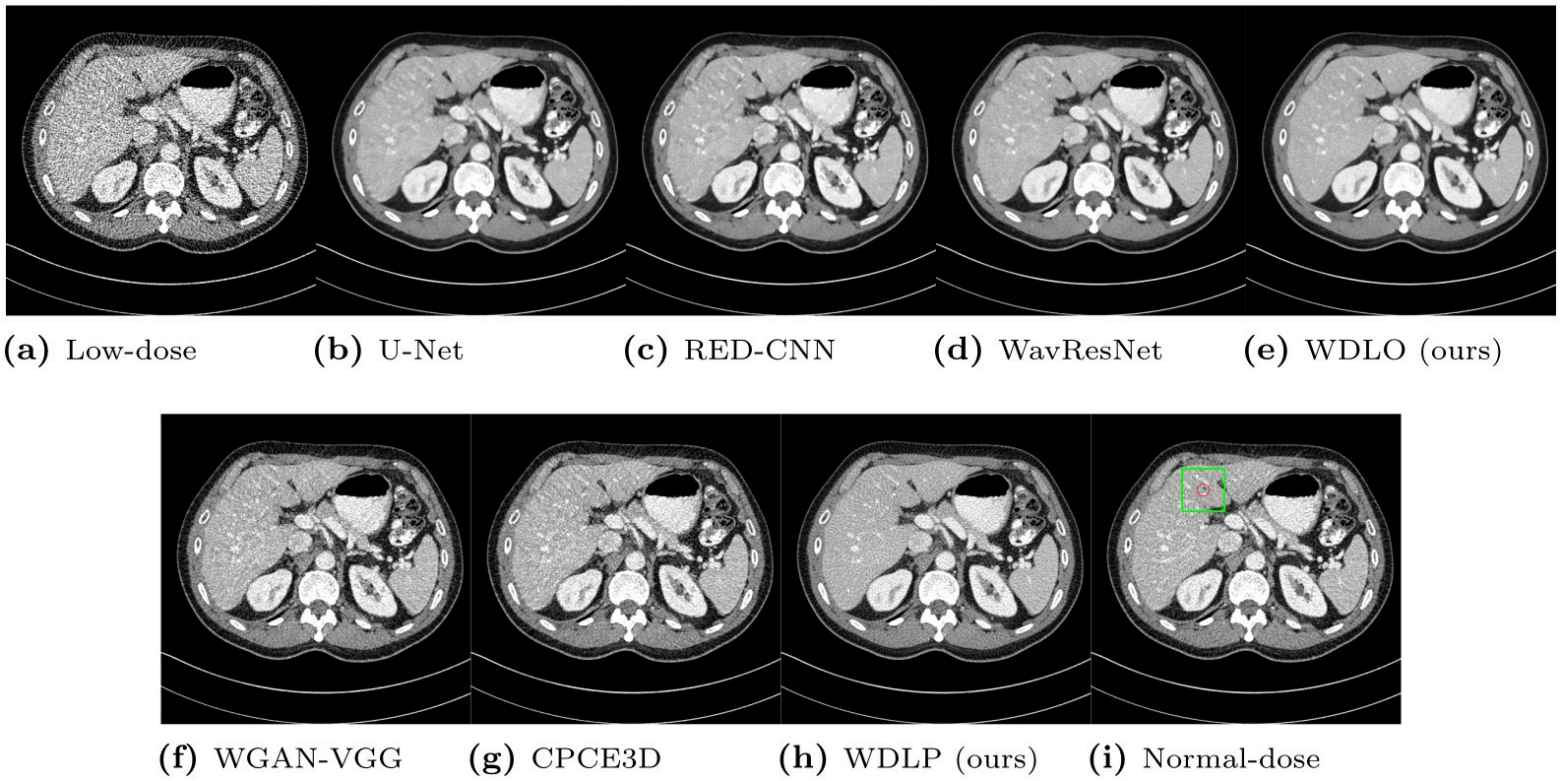
Representative slice of the abdomen from the Mayo Clinic dataset. The display window is [-150, 250]HU.

**Fig 11:**
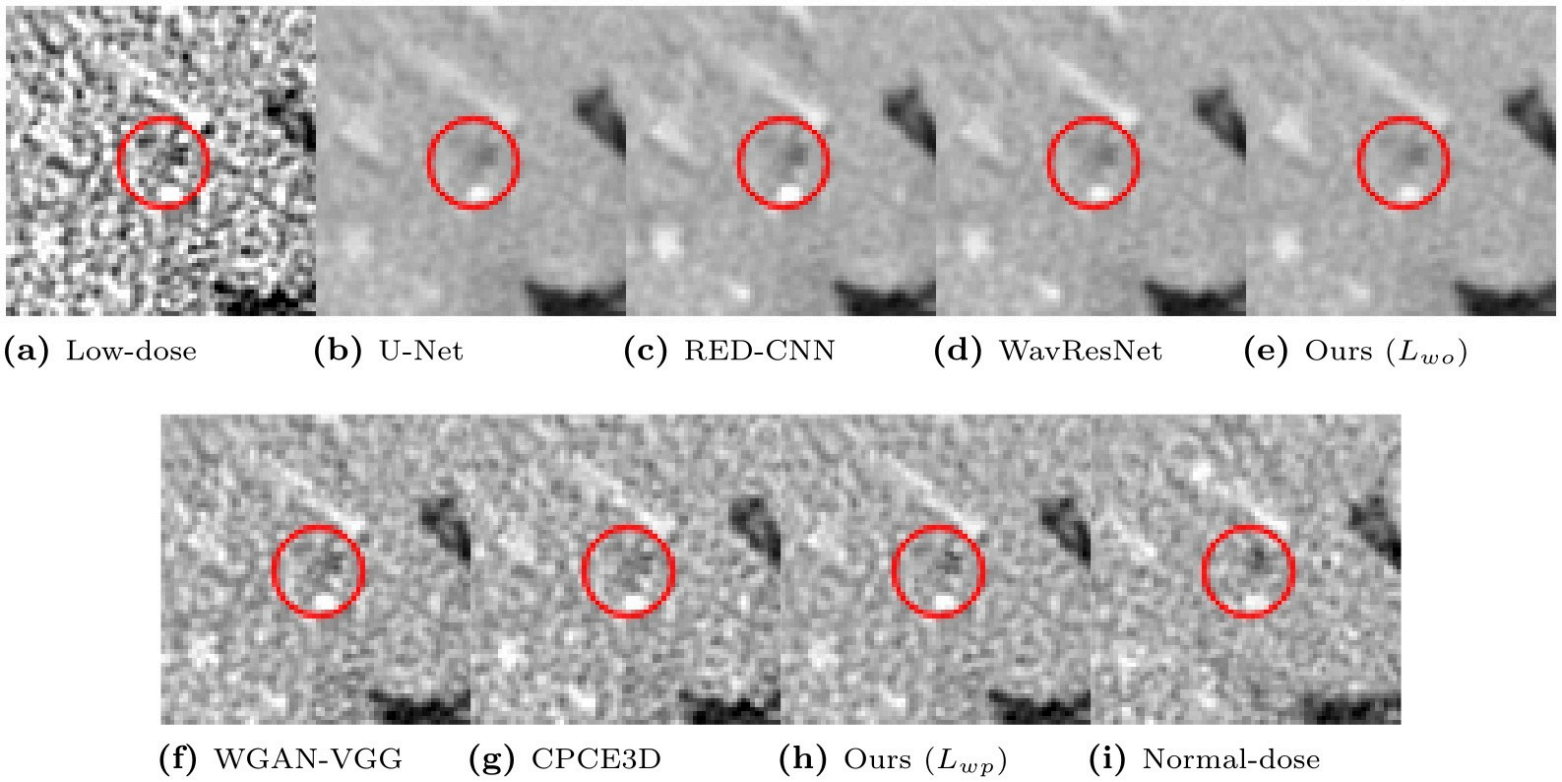
Regions of interest from Fig 10. The display window is [-150, 250]HU.

**Fig 12:**
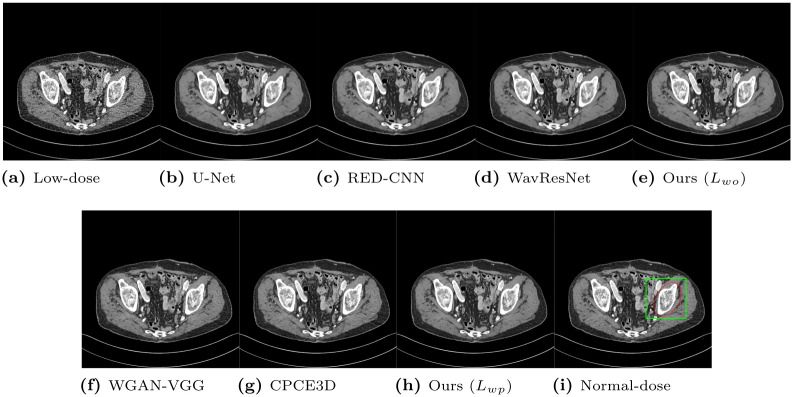
Representative slice of the pelvis from the Mayo Clinic dataset. The display window is [-160, 240]HU.

**Fig 13:**
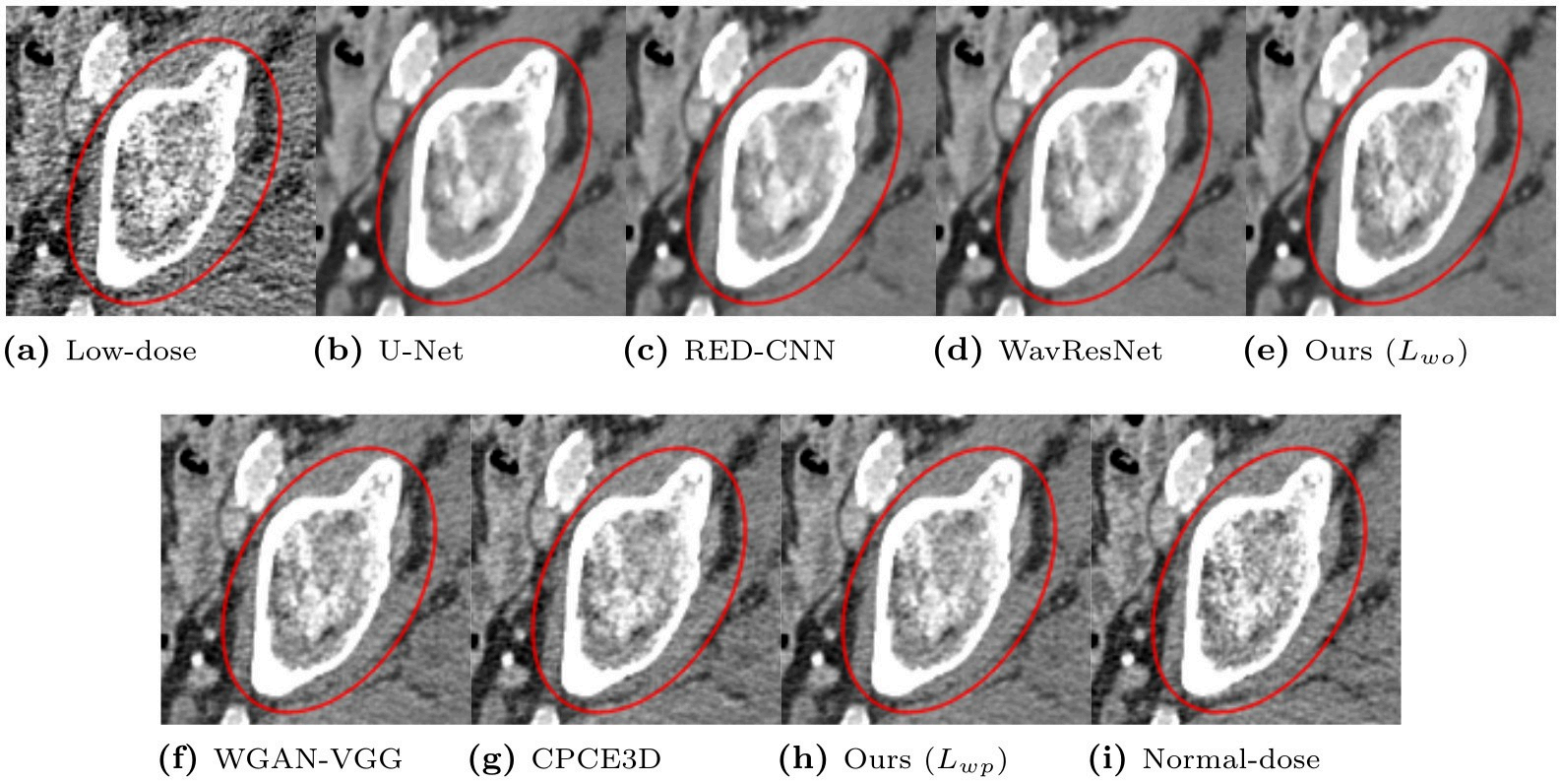
Regions of interest from Fig 12. The display window is [-160, 240]HU.

From the Mayo Clinic dataset, we selected CT slices that contain lesions and bone tissues. Representative CT slices that contain a lesion are depicted in Figs 10 and 11. Perception of lesions, which can be understood as visibility, should not be degraded after producing denoised CT images from the proposed algorithms. As expected, our network with the optimization of *L*_*wo*_ removed the noise better than the other algorithms, but the shapes are smoothed, which weakens the contrast of the lesions in the region of interest. The networks optimizing perceptual loss (WGAN-VGG, CPCE3D, and our network with optimizing *L*_*wp*_) recovered the loss of the contrast, and the visibility was strengthened. Another CT slice that contains bone tissues is depicted in Figs 12 and 13. Bone tissues are a good indicator of the sharpness and edges because they have a very subtle but complicated texture pattern. Among networks that minimize objective loss, our network with *L*_*wo*_ also has a more accurate texture pattern with relatively less loss of the trabecular microstructure than the others, which indicates that perceptual quality can be more easily enhanced from the proposed network. In addition, WGAN-VGG, CPCE3D, and the proposed network with the optimization of *L*_*wp*_ exhibit comparable perceptual quality to the reference normal-dose CT image. They all have similar texture patterns, and the details are slightly lost from the high- dose CT image.

Moreover, our proposed network with *L*_*wp*_ has a higher objective quality with less noise than WGAN-VGG and CPCE3D. This result is significant because we achieved higher noise reduction performance than the WGAN-VGG and CPCE3D, even when we maximized the perceptual quality to be comparable to the WGAN-VGG and CPCE3D. Thus, our proposed network demonstrated a better PD trade-off than the current state-of-the-art methods.

#### 3.2.4 Blind reader study with radiologists

To conduct a blind reader study, we selected a representative group of 10 denoised CT slices from LDCT denoising algorithms. Seven CT slices are from the Mayo Clinic dataset, and the remaining three CT slices are from phantom datasets. Reference normal-dose and low-dose images are included in each group, and we randomly showed our denoised CT images to two radiologists with more than 10 years of experience in chest CT interpretation. They were asked to score each image with the following criteria: noise reduction, structural preservation, and overall quality. The score ranged from 1 (unacceptable) to 5 (excellent), and the resulting scores for each algorithm are reported as the mean score of two radiologists plus or minus the standard deviation (*mean*±*std*) in Table 7.

**Table 7:** Subjective image quality scores (*mean*±*std*) for different algorithms from a blind reader study.

Algorithm	Noise reduction	Structural preservation	Overall quality
Normal-dose	-	-	4.00 ± 0
Low-dose	-	-	1.15 ± 0.37
RED-CNN	4.47 ± 0.52	3.57 ± 0.42	3.58 ± 0.47
WavResNet	4.55 ± 0.27	3.71 ± 0.35	3.70 ± 0.45
U-Net	4.48 ± 0.52	3.60 ± 0.41	3.60 ± 0.45
Ours (*L*_*wo*_)	**4.68 ± 0.25**	3.77 ± 0.24	3.81 ± 0.32
WGAN-VGG	3.77 ± 0.31	3.70 ± 0.24	3.74 ± 0.23
CPCE3D	3.82 ± 0.57	3.83 ± 0.22	3.88 ± 0.36
Ours (*L*_*wp*_)	4.12 ± 0.27	**3.88 ± 0.21**	**4.09 ± 0.35**

In general, algorithms optimizing objective loss (RED-CNN, WavResNet, U-Net and ours with *L*_*wo*_) have excellent noise reduction performance, and algorithms pursuing perceptual loss (WGAN-VGG, CPCE3D, and ours with *L*_*wp*_) received excellent scores in terms of structural preservation and overall quality. Our proposed network with *L*_*wo*_ optimization achieved the best performance in noise reduction, and our network with *L*_*wp*_ optimization performs the best performance in structural preservation and overall quality. Interestingly, ours (*L*_*wo*_) scored a higher score in structural preservation and overall quality than WGAN-VGG [13] although ours (*L*_*wo*_) optimized the objective quality whereas WGAN-VGG optimized perceptual quality.

#### 3.2.5 Perception-distortion trade-off curve

**Fig 14:**
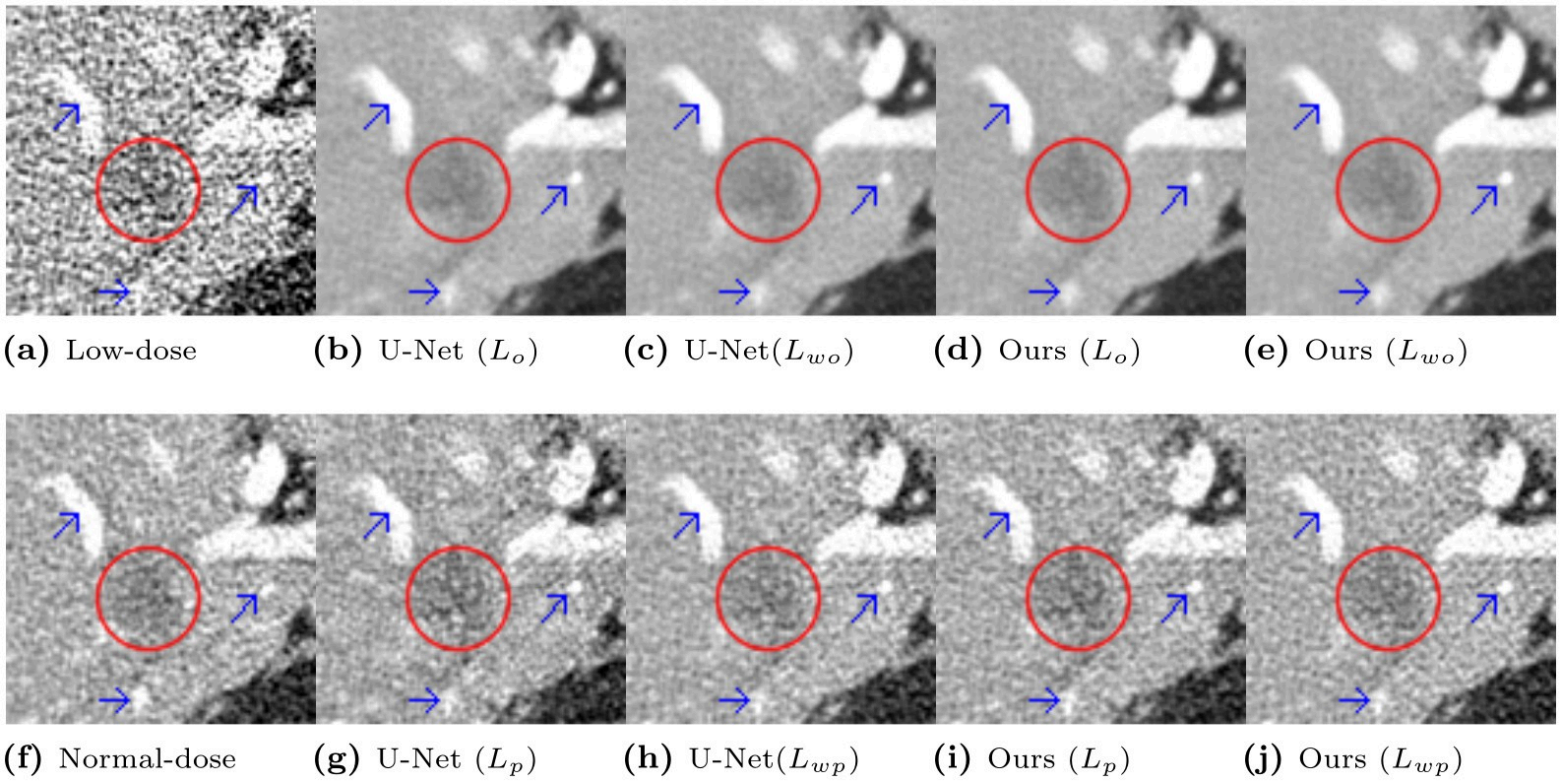
Denoised CT images from U-Net and the proposed methods with various loss functions. Images in (b), (c), (d), and (e) are from the networks that optimized the objective loss functions. Images in (g), (h), (i), and (j) are from the networks that optimized the perceptual loss functions. The display window is [-160, 240]HU. Red circles and blue arrows point to the low attenuation lesion and blood vessels in the posterior right liver lobe, respectively.

To prove that our proposed algorithms have a more effective PD trade-off, we implemented two networks, U-Net [19] and the proposed network, and optimized two networks with different loss functions: *L*_*wo*_, *L*_*wp*_, *L*_*o*_, and *L*_*p*_ in (10), (19), (20) and (21), respectively. Their objective quality results are summarized in Table 8. Their representative whole cropped CT slices for effective perceptual quality are depicted in are in Fig 14.

**Table 8:** Trade-off of perception-distortion of U-Net and our proposed network. The metric is the peak signal to noise ratio (PSNR).

		U-Net	Ours
Loss	*L*_*o*_	39.677	39.875
	*L*_*p*_	37.717	38.856
	*L*_*wo*_	39.695	39.950
	*L*_*wp*_	39.013	39.110
Trade-off	*L*_*o*_ − *L*_*p*_	1.960	1.019
	*L*_*wo*_ − *L*_*wp*_	0.682	0.840

From Table 8, from the point of objective quality, reveals the following:

- We achieved a higher PSNR with the optimization of *L*_*wo*_ than the optimization of *L*_*o*_ in both networks: 39.677 < 39.695 (U-Net) and 39.875 < 39.950 (ours).
- Optimization with *L*_*wp*_ had a higher PSNR than the optimization with *L*_*p*_ in both networks: 37.717 < 39.013 (U-Net) and 38.856 < 39.110 (ours).
- The PD trade-off from the optimization with *L*_*wo*_ to the optimization *L*_*wp*_ is smaller than from the optimization with *L*_*o*_ to the optimization *L*_*p*_: 0.682 < 1.960 (U-Net) and 0.840 < 1.019 (ours).

The CT images in the top row of Fig 14, which are the output of the network optimizing the objective loss, exhibit higher noise reduction, but the marked areas show overly smoothed results for shape. In contrast, the network optimizing the perceptual loss generated images with sharper edges and higher contrast, displayed in the bottom row of Fig 14. The proposed network with optimization of *L*_*wp*_ has a higher PSNR than the others in the bottom row. Therefore, it has a lower noise level in our denoised results and low distortion compared to the ground truth image. From these facts, the wavelet perceptual loss (*L*_*wp*_) effectively improves the trade-off between the PD relationship. Thus, we minimize the loss of the objective quality while maximizing the perceptual quality.

**Fig 15:**
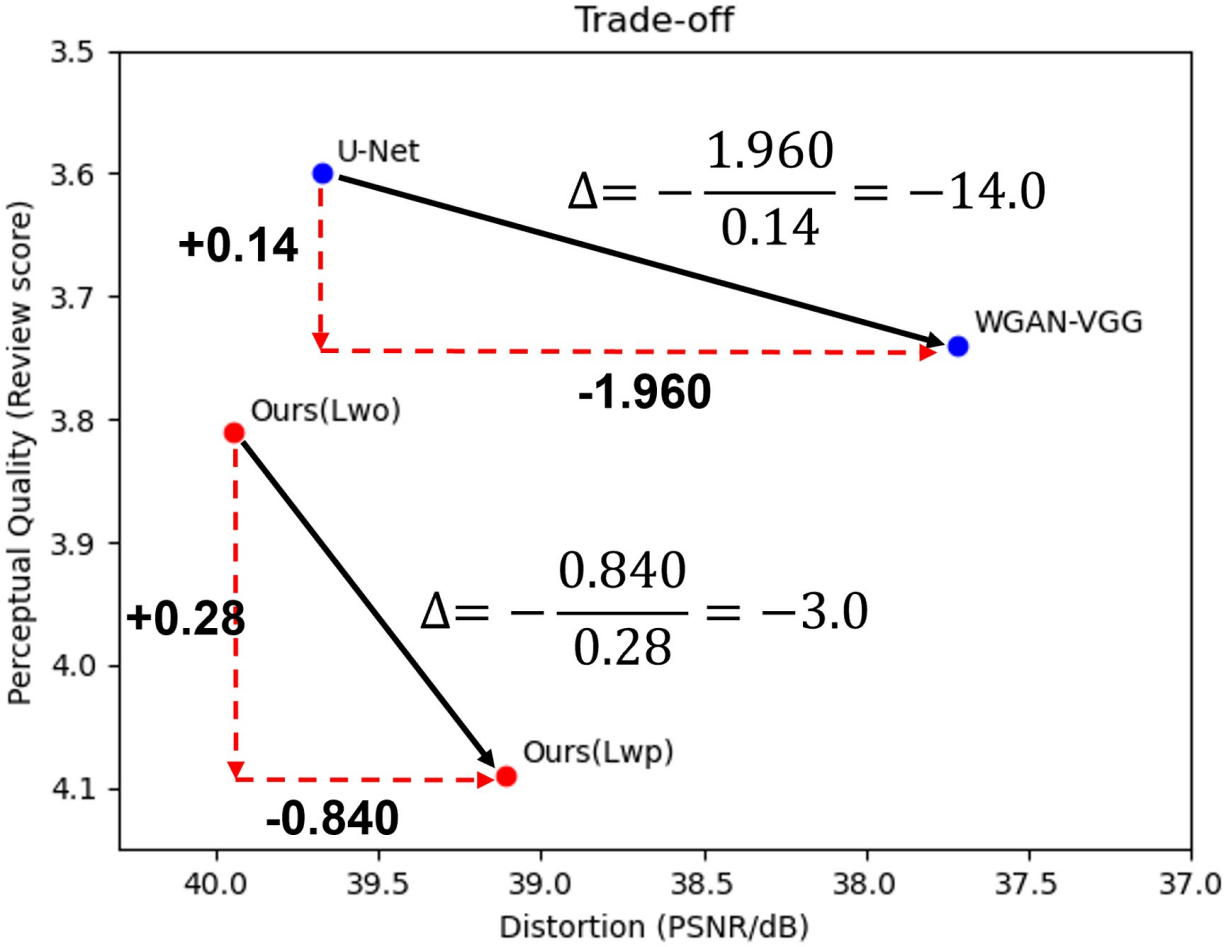
Trade-off of U-Net between the objective and perceptual loss optimization and ours between the objective and perceptual loss optimization in wavelet transform loss domain.

To provide an example of the PD trade-off using our blind study results, we chose the U-Net, WGAN-VGG, and the proposed networks with optimization of *L*_*wo*_ and *L*_*wp*_ and depicted the trade-off in Fig 15. Because we trained WGAN-VGG with U-Net, it is a network that optimized the perceptual quality from the U-Net network. In the case of U-net in Fig 15, in order to raise the perceptual quality from 3.60 to 3.74, the objective quality decreased from 39.68 to 37.71. For our proposed algorithm, to improve the perceptual quality from 3.81 to 4.09, the objective quality was reduced from 39.95 to 39.11. Here, the value obtained by dividing the decrease in objective quality by the increase in perceptual quality can be interpreted as a trade-off value of objective quality to increase the unit perceptual quality value, and the resulting division values of U-Net and our proposed algorithm are -14.0 and -3.0, respectively. Although the blind review score is subjective to radiologists, this measure demonstrates that the proposed methods with the wavelet domain loss have a better trade-off than the U-Net-based network between the optimization of *L*_*o*_ and *L*_*p*_.

## 4 Discussion

In this paper, we proposed a novel LDCT denoising method to generate high objective and perceptual quality denoised images. Moreover, recent studies have focused more on the perceptual quality in LDCT denoising; however, the objective quality is still an important key factor to measure algorithm performance. Thus, our motivation for this paper was to maintain the objective quality to be as high as possible while enhancing the perceptual quality. Our key contributions to accomplish this goal are as follows. 1) We developed the network with the SWT, which can achieve the highest objective quality among the state-of-the-art denoising algorithms for natural and LDCT images. 2) We also presented a novel wavelet subband-specific learning strategy to preserve the structural and textural information in images while minimizing the loss of the objective quality. As a result, we demonstrated a better PD trade-off with the proposed method using the wavelet domain loss. Finally, 3) we tested the performance of the proposed methods with a phantom dataset and the *NIH-AAPM-Mayo Clinic Low Dose CT Grand challenge dataset*, demonstrating that ours can achieve better objective quality while preserving the perceptual quality than other state-of-the-art LDCT denoising methods.

The lack of proper metrics for measuring perceptual quality in an LDCT denoising task made it difficult to evaluate the perceptual quality of the algorithms assessed in this paper. To evaluate the perceptual quality, we invited experienced radiologists to conduct a blind reader study. However, conducting a blind reader study is very time-consuming and expensive, and the outcome could depend on the radiologists’ experience [48]. As future work, we plan to develop a metric for perceptual image quality that is well correlated with the human visual system’s characteristics in evaluating LDCT image quality. If such a metric were developed, it would be expected to evaluate the perceptual image quality at a low cost with little time investment.

As the network is trained using one-to-one mapping from low-dose to normal-dose CT images and normal-dose images are not often clean images with no noise, our proposed networks might learn the residual noise of the target normal-dose CT images. In addition, as normal-dose CT images are set as the standard of the network performance measurement, the networks cannot generate a denoised image that can exceed the quality of the normal-dose CT images. According to the overall quality reported in Table 7, WGAN-VGG and CPCE3D were slightly lower than the normal-dose image score value, and only our algorithm was higher than the normal-dose image score value with a marginal difference. This outcome is a very common problem in LDCT denoising algorithms, but it does not seem to be a problem that cannot be overcome. For instance, one could create a network capable of deriving an image superior to the original reference image by integrating unsupervised learning into the essential supervised learning-based LDCT denoising problem [49].

Until recently, most LDCT denoising has focused on post-processing denoising due to the inability to access 2D projection data or proprietary reconstruction software. However, this post-processing method in the image domain has a disadvantage in that it cannot effectively suppress noise or artifacts that have already been introduced in the process of reconstructing the projection images to the 3D CT images with filtered back projection [50]. Recently, research results on reconstructing 2D images into 3D images using neural networks have been published [51]. As other future work, we plan to optimize these reconstruction networks for the denoising task incorporating 2D projection images to build an even better denoising model. Moreover, as volumetric CT images have 3D spatial information, we can employ spatial information in the out-of-plane directions to further enhance our denoising networks [15, 17].

Last but not least, due to the difficulty to obtain pairs of low- and normal-dose CT images, researches on unsupervised and self-learning based denoising is being more actively conducted. CycleGAN [25] to translate from noisy to clean CT images was successfully applied to LDCT denoising [24, 52]. Self-learning-based models using only noisy images have been proposed in LDCT denoising tasks. Studies [22, 23, 53–55] combined self-learning strategy with a CT reconstruction pipeline or a physics-based noise model. Noise2Context [56] and Noise2Neighbors [57] effectively suppressed noise with a physics-based CT model. Although these unsupervised and self-learning models successfully reduced noises, they still have large margin to follow with the state-of-the-art supervised LDCT denoising models [3, 5, 13, 15, 19]. Furthermore, as they still have focused more on noise reduction, models to enhance perceptual quality [13, 58] or our work can be combined with these unsupervised or self-learning LDCT denoising models to secure denoised CT images with both excellent objective and perceptual quality.

## 5 Conclusion

In conclusion, the studied networks optimizing the objective loss exhibited excellent performance in suppressing noise at the cost of the loss in detailed textures and edges that are important for clinical diagnosis. In contrast, the networks optimizing the perceptual loss resulted in relatively high noise in generating realistic CT images with high perceptuality. With the key insight that high- and low-frequency components in an image have different characteristics, we proposed a novel network capable of achieving high objective and perceptual quality using the presented frequency subband-specific loss in the wavelet domain. Our proposed methods demonstrate the effective PD trade-off in LDCT denoising. With phantom and clinical datasets, our proposed methods result in an accurate and realistic CT image and achieve better performance than the existing state-of-the-art methods in terms of objective and perceptual quality.
